# *Drosophila* RSK: A Pivotal Regulator of Circadian Plasticity at the Neuronal and Behavioral Level

**DOI:** 10.1177/07487304261434715

**Published:** 2026-04-12

**Authors:** Athira Theyyassanchery Mani, Vivian Backs, Christian Werner, Charlotte Helfrich-Förster, Thomas Raabe

**Affiliations:** * Molecular Genetics, Biocenter, University of Würzburg, Würzburg, Germany; † Biotechnology and Biophysics, Biocenter, University of Würzburg, Würzburg, Germany; ‡ Neurobiology and Genetics, Biocenter, University of Würzburg, Würzburg, Germany

**Keywords:** Drosophila, clock neurons, structural plasticity, circadian rhythm, adaptation

## Abstract

Circadian neuronal plasticity describes daily recurring changes at the level of neuronal morphology, connectivity and synaptic processes. Disturbance of these plastic changes could result in inflexibility of an organism to adapt behavior to changing environmental cues. The mitogen activated protein kinases (MAPK)/ERK signaling pathway is involved both in circadian processes and neuronal plasticity. Ribosomal S6 kinases (RSK) act as downstream mediators of ERK signaling with apparently pleiotropic—but sometimes poorly understood- functions in the nervous system. This is illustrated by some major gaps in our understanding of the pathophysiological processes caused by RSK2 mutations in humans that lead to intellectual disabilities. Previous studies described the role of *Drosophila* RSK as one regulator of the molecular circadian oscillator. Here we could show that RSK kinase activity is required to control another aspect of circadian rhythmicity, the daily remodeling of the dorsal branching pattern of the small ventral lateral neurons (s-LN_v_) as the central pacemaker cells. Loss of RSK function resulted in more fasciculated and less branched s-LN_v_’s in the early morning, which could affect synaptic in- or output connectivity. Increased fasciculation correlated with a reduced number of Bruchpilot sites as a marker for presynapses. Analysis of the expression of the Pigment Dispersing Factor PDF in s-LN_v_’s, the most important signaling factor between clock neurons, revealed no evidence of changes in *RSK* mutants. Consistent with unaffected PDF signaling as a major output from the s-LN_v_’s, *RSK* mutant flies are rhythmic. Their free-running rhythms show even a significantly higher power than those of the wild-type controls. This robustness is at the expense of flexibility to adapt their activity to variations in light conditions. Together with the known role of RSK in olfactory learning and memory processes our results suggest that RSK is required to maintain experience dependent plasticity.

Circadian clocks constitute an endogenous timekeeping system that allows organisms to synchronize behavior and physiology to daily recurring environmental changes, mainly the dark light cycle, but also other non-photic factors such as temperature, social interactions and food availability (Young, 2018). Anticipation and adaptation are critical features of this system. Given its central role at the interface between environment and organismal functions, it is not surprising that disturbance of the circadian clock is associated with a variety of diseases, including mental health problems and metabolic dysfunction (Jagannath et al., 2017; Panda, 2016; Patke et al., 2020).

At the molecular level, circadian oscillations are generated in so-called clock cells by several interconnected transcriptional-translational feedback-loops. Although many tissues and organs house circadian clocks, the master clock system resides in the brain; the suprachiasmatic nucleus (SCN) in mammals and several groups of clock neurons in *Drosophila melanogaster* (Dubowy and Sehgal, 2017; Hegazi et al., 2019; Patke et al., 2020). The 240 clock neurons in the *Drosophila* brain (Reinhard et al., 2024) are subdivided into three clusters of dorsal neurons (DN1-3) and several groups of lateral neurons, which include lateral posterior neurons (LPNs), dorsolateral neurons (LN_d_’s) and two groups of ventrolateral neurons (large (l-)LN_v_’s and small (s-)LN_v_’s). To generate rhythmic behaviors, the different clock neurons need to communicate with each other by complex network of forward and feedback signals (Helfrich-Förster and Reinhard, 2025; Top and Young, 2018). Among these is the neuropeptide pigment-dispersing factor (PDF), an essential paracrine signal within the clock network and a key output signal of the clock, which is expressed in both groups of LN_v_’s except for the fifth s-LN_v_ (Helfrich-Förster, 1995; Renn et al., 1999). The s-LN_v_’s are considered as master pacemakers because they set the rhythm for other clock neurons and drive rhythmic activity in constant darkness (Grima et al., 2004; Rieger et al., 2006; Stoleru et al., 2005). Another remarkable feature of s-LN_v_’s is the circadian remodeling of their branched neurite terminals in the dorsal brain, with maximum complexity in the morning (Fernandez et al., 2008), which correlates with changes in synaptic contacts throughout the day (Gorostiza et al., 2014; Ispizua et al., 2025). Together with circadian remodeling of other clock neurons, this fine-tunes communication within the clock network and adapts behavior to changes in environmental conditions (Duhart et al., 2020; Fernandez et al., 2020; Song et al., 2021). Structural circadian plasticity has also been observed in the SCN and other brain regions in vertebrates such as the hippocampus, providing a potential link to time-of-day depending learning and memory processes (Bosler et al., 2015; Krzeptowski et al., 2018; Snider et al., 2018).

The molecular mechanisms that drive structural remodeling of s-LN_v_’s remain incompletely understood. Among the molecules identified are PDF processed by the matrix metalloproteinase Mmp1 (Depetris-Chauvin et al., 2014), the small GTPase Rho1 as a regulator the cytoskeleton (Petsakou et al., 2015), the cell adhesion molecule Fasciclin 2 (Sivachenko et al., 2013), Unc5 as an axon guidance protein (Fernandez et al., 2020), proteins of the autophagosome (Szypulski et al., 2024) and microRNAs (Cusumano et al., 2018; Nian et al., 2019).

Reversible phosphorylation by protein kinases is another potential mechanism to regulate neuronal remodeling in a time-dependent manner. In particular, it is known that the mitogen-activated protein kinase (MAPK)/ERK signaling pathway influences both circadian processes and synaptic plasticity, with dysregulation contributing to neurological developmental disorders, behavioral and cognitive dysfunctions (Goldsmith and Bell-Pedersen, 2013; Lodovichi and Ratto, 2023; Snider et al., 2018; Thomas and Huganir, 2004; Borrie et al., 2017).

Among the many downstream effectors of ERK are Ribosomal S6 kinases (RSKs), a family of serine/threonine kinases with four RSK isoforms (RSK1-4) present in vertebrates and a single isoform (RSK) in *Drosophila*. RSK proteins contain two kinase domains: the N- terminal kinase domain (NTKD) and the C-terminal kinase domain (CTKD) separated by a linker region. Binding of ERK stimulates CTKD activation, which in turn is required for NTKD activation (Romeo et al., 2012). The NTKD is considered as the main effector kinase for phosphorylation of downstream targets, however, NTKD or even complete kinase independent functions were described in *Drosophila* during eye development and in the circadian system (Beck et al., 2018; Kim et al., 2006; Tangredi et al., 2012). In humans, loss of RSK2 causes Coffin-Lowry syndrome, which is associated with severe mental impairments (Pereira et al., 2010). *RSK2* knock-out in rodents affects a variety of cognitive and emotional behaviors, which might be a consequence of impaired adult neurogenesis, altered synaptic spine morphology, changes in synaptic transmission and up-regulated ERK signaling because of missing negative feedback inhibition (Fischer and Raabe, 2018). Similar, in *Drosophila*, loss of RSK function results in altered ERK activity, a decrease in the number of synaptic sites at motoneuron endings, impaired anterograde axonal transport, operant, associative and spatial learning deficits and a short period phenotype in constant darkness (Fischer and Raabe, 2018). Specifically, the short-period phenotype results from alteration of the circadian oscillator and has been attributed at least in part to missing phosphorylation and negative regulation of the kinase Shaggy (SGG, vertebrate GSK3β) in s-LN_v_’s (Beck et al., 2018; Akten et al., 2009). SGG acts as key regulator for timely nuclear entry of Timeless and Period for feedback inhibition their own transcription. To fulfill its function in the molecular clock, cyclic RSK protein expression is not required (Akten et al., 2009), suggesting that RSK kinase activity is modulated in a time-dependent manner by ERK-mediated signal transduction or by yet unknown upstream regulators.

In this study, we examined the effects of RSK loss-of-function and domain-specific perturbations on s-LN_v_ dorsal arborization, synaptic number and circadian locomotor rhythms. We show that RSK is required for circadian s-LN_v_ remodeling. Maximal arborization complexity and synapse number in the morning were decreased in *RSK* mutants. Conversely, constitutive activation of RSK forces s-LN_v_ to increase branching in the early night, when complexity is minimal in wild-type flies. At the behavioral level, *RSK* mutant flies show stronger rhythms but adapt slower to changes in light-dark conditions than wild-type flies. Our findings imply that RSK is required for circadian structural adaptation and behavioral flexibility.

## Materials and Methods

### Fly Stocks and Genetics

Flies were maintained at 25°C on cornmeal food in a 12:12-hour light-dark (LD) cycle except for long- and short-day experiments. Canton Special (CS) was taken as the control. The *RSK* null allele was obtained from the Bloomington Drosophila stock center (BDSC #81108) and resulted from replacing the complete *RSK* coding region by CRISPR-mediated HDR with a cassette carrying the lox-P flanked 3xP3-RFP marker. This mutation was first brought into a CS background and then the *3xP3-RFP* cassette was removed by Cre recombinase-mediated excision to obtain *RSK*^⊿^, which after sequence verification was used in all experiments. The morphology of s-LN_v_’s was visualized by expression of *UAS-mCD8::GFP* (BDSC #5130) with *PDF-Gal4* (kindly provided by Jeffrey C. Hall). To ensure that mCD8::GFP has no adverse effects on the function of PDF neurons, we expressed it in these cells and recorded the activity rhythms of the flies under long and short days as well as under constant darkness. We found no differences in behavior compared to the control flies (C.H.-F., unpublished). Since circadian behavior is very sensitive to disturbances in PDF neurons, we are confident that these neurons function normally even with mCD8::GFP in their membranes. For stochastic labeling of single s-LN_v_’s, the MultiColor FlpOut technique (MCFO) was applied (Nern et al., 2015). *P{10xUAS(FRT .stop)myr::smGFP-OLLAS}, PBac{10xUAS(FRT.stop)myr::smGFP-HA}, P{10xUAS(FRT.stop)myr::smGFP-V5-THS-10xUAS(FRT.stop)myr::smGFP-FLAG}* (BDSC-#64091) was expressed with *PDF-Gal4* in combination with *P{hs-FLPG5}* (BDSC #55816) in control or *RSK*^⊿^ flies. Stochastic removal of the FRT stop cassettes was induced by a 20-minute heat-shock at 37°C. After 2 days, brains were dissected and immuno-stained with anti-FLAG, anti-HA and anti-V5 antibodies.

For the generation of transgenic flies expressing RSK, we subcloned the complete open reading frame from a previously described *pAC5.1-Myc::RSK* construct (Beck et al., 2018) by linker PCR into the pUAST.attB vector (Bischof et al., 2007). To generate mutated variants of RSK, the *pAC5.1-Myc::RSK* construct was modified by in vitro mutagenesis to introduce kinase activating amino acid substitutions into the NTKD (S357E, SE) or CTKD (T732E, TE). Similar, kinase dead versions for the NTKD (K231M, KM) and the CTKD (K597M, KM) were generated. The following combinations were subcloned into pUAST.attB and verified by sequencing: *RSK*^SE,KM^, *RSK*^KM,TE^, *RSK*^SE,TE^, *RSK*^KM,KM^ transgenic flies were generated by PhiC31 -mediated integration into the third chromosomal attP landing site of fly strain ZH-86Fb loxP (FlyORF Injection Service, Zürich, CH).

### Immunohistochemistry

For immunostainings, experiments were done at time points ZT 2 and ZT 14. All flies were directly fixed in 4% paraformaldehyde in PBS (10 mM Na_2_HPO_4_, 1.8 mM KH_2_PO_4_, 2.7 mM KCl, 137 mM NaCl) supplemented with 0.15% Triton X-100) for 2.5 hours and then washed in PBT (PBS plus 0.3% Triton X-100, used for all washing steps). After blocking (PBT supplemented with 5% normal donkey serum) for 1 hour, samples were incubated overnight at 4°C with combinations of the following primary antibodies: mouse anti-Bruchpilot (1:30, clone nc82, kind gift from E. Buchner, Würzburg), mouse anti-FLAG-tag (1:500, clone M2, Merck), chicken anti-GFP (1:1500; #ab13970, abcam), rabbit anti-GFP (1:1000, #A6455, Thermo Fisher Scientific), rabbit anti-HA-tag (1.800, #3724, Cell Signaling Techn.), mouse anti-PDF (1:500, clone c7, Developmental Studies Hybridoma Bank), chicken anti-V5-tag (1:100, #A190-118a, Thermo Fisher Scientific),. Secondary antibodies were AlexaFluor 488, Cy3 or Cy5-conjugated (Dianova). Embedding of brains was done using Vectashield (Vector Laboratories).

### Confocal Imaging and Structured Illumination Microscopy

s-LN_v_ arborizations were imaged with a Leica SP8 confocal microscope using 20× or 63× oil immersion objectives. Z-stacks were acquired at 0.2 µm intervals, with resolution set to 1024 × 1024 px and higher. The same laser and detector settings were used for all samples in a given experiment. Structured Illumination Microscopy (SIM) imaging was performed using a Zeiss Elyra 7 system equipped with ZEN software and applying the SIM^2^ algorithm. Samples were imaged with a 63×/1.40 NA oil immersion Plan-Apochromat objective, using laser excitations at 488 nm and 561 nm. Fluorescence signals were collected over 13 phase shifts and recorded on a PCO Edge 4.2 M sCMOS camera passing adequate emission filters. ZEN Black software was used for SIM image reconstruction (Carl Zeiss Microscopy). Tetraspeck (Invitrogen) beads were recorded in z-stacks to correct chromatic aberration using affine transformations.

### Quantification of s-LN_v_ Arborization

Analysis of the 3D spread was carried out with MorphoScope, a Python-based graphical user interface developed by Francisco Joaquín Tassara (2025), which implements the methodology originally published by Blau and colleagues (Petsakou et al., 2015). The source code and documentation are available at https://github.com/FranTassara/MorphoScope. Images stacks were processed with the ImageJ distribution Fiji (Schindelin et al., 2012) and projections were traced and reconstructed in three dimensions to quantify total projection spread volume. All analysis were performed blind to genotype.

### Quantification of BRP Puncta

For labeling the presynaptic protein Bruchpilot (BRP) specifically in s-LN_v_’s, the synaptic tagging with recombination (STaR) method was used (Chen et al., 2014). Briefly, the endogenous *BRP* genomic sequence in a BAC was modified with a FRT stop cassette followed by the GFP sequence. Combing this fly line (*PBac{brp(FRTstop)GFP}*, BDSC-#56507) with *UAS-Flipase* (BDSC #8209) and *PDF-Gal4* resulted in excision of the FRT cassette and expression of BRP::GFP in s-LN_v_’s (and other PDF positive neurons). After SIM microscopy, the region of interest was delineated by generating a masked channel for the PDF staining using the Imaris Channel Tool. This mask then served as a template to extract BRP puncta adjacent to PDF-positive regions. Using the Imaris software, the numbers of BRP puncta were quantified by selecting puncta with diameters ranging from 0.0626 to 0.235 µm within the region of interest. Based on PDF signals, the overall volume of the terminals was determined and used to calculate the density of BRP puncta. Thresholds were applied based on absolute intensity (A.U.). For BRP puncta, a threshold of 1500 A.U. was used, whereas quantification of PDF signal volume was calculated using a threshold of 4000 A.U.

### Quantification of PDF Expression

To analyze *pdf* gene expression, total RNA was extracted from 25 heads from flies at ZT 2 or ZT 14 using the Trizol reagent (Invitrogen, ThermoFisher Scientific) and then reverse transcribed with the High Capacity cDNA Synthesis Kits (Applied Biosystems, ThermoFisher Scientific). RT-qPCR was done using PowerUp SYBR Green Master Mix (Applied Biosystems) on a StepOnePlus (Applied Biosystems) real-time thermal cycler. Results were expressed as fold change in expression of the treated sample in relation to untreated samples and relative to the reference gene *rp49*. Mean ± SEM was calculated based on at least three replicates from each of the 4 to 5 independent biological samples per genotype at ZT 2 or ZT 14. The following primers were used to amplify the cDNA of target genes:

*pdf* forward: 5′-CTATGTGCGCAAGGAGTACAATCG-3′,
*pdf* reverse: 5′-GCATCGTTCATGTTCTTGGGCAG-3′,
*rp49* forward: 5′-GCCCAAGATCGTGAAGAAGC-3′,
*rp49* reverse: 5′-CGACGCACTCTGTTGTCG-3′.

To quantify PDF peptide expression levels, brains from males of the genotype *PDF-Gal4; UAS-mCD8::GFP* and *RSK*^⊿^; *PDF-Gal4; UAS-mCD8::GFP* (both in CS background) were stained for GFP and PDF and imaged by confocal microscopy. A binary mask was created in the GFP channel using a self-written macro in Fiji/ImageJ (kindly provided by Nils Reinhard). Prior to this, the automated thresholding method “Otsu” was selected and applied to all images. The binary mask was superimposed on the respective image file and PDF intensities were quantified in the ROI with a second self-written Fiji/ImageJ macro. For background correction, the averaged signal intensity from three representative areas outside of the ROI was subtracted.

### Locomotor Activity Recordings and Analysis

Locomotor activity of individual male flies (starting 0-2 days after eclosion) was recorded using the *Drosophila* Activity Monitor (DAM) system (TriKinetics). During recording, flies were provided with a medium of agar and sucrose as food source. The monitors were placed in boxes which were kept at a constant temperature of 25°C. White LEDs provided light intensity of 500 lux. The number of beam crosses of individual flies in intervals of 1 minute was counted, and data was collected using the DAM System 2.1.3 software. All flies were first entrained for 5 days in a 12:12-hour LD cycle followed by constant darkness (DD) for 10 to 15 days or altered LD conditions (phase shifts, long and short days). Individual actograms and periodograms were generated with the help of ImageJ plugin ActogramJ (Schmid et al., 2011), and period length and rhythm power during DD were determined by the embedded Lomb–Scargle periodogram analysis. Average actograms for wild-type flies and *RSK*^⊿^ mutants were calculated from the individual actograms and plotted graphically, while the individual periodograms of wild-type flies and *RSK*^⊿^ mutants were combined in a single plot to show the distribution of period and rhythm power for the two groups. Average activity profiles during LD were created as described (Schlichting and Helfrich-Förster, 2015). Phases of morning and evening activity peaks, peak heights, and slopes from the start to the maxima of morning and evening activity were calculated for each individual fly using an Excel Macro (Yokosako et al., 2025) kindly provided by Taishi Yoshii (Okayama University, Japan) that uses the same calculations as described (Persons et al., 2022), with a single exception concerning the slopes from the start to the maximum of morning and evening activity.

### Data Analysis

Data were tested for normality using the Kolmogorov-Smirnov and Shapiro-Wilk tests. Non-normally distributed variables were analyzed using the Mann-Whitney U test (two groups) or Kruskal-Wallis test (>2 groups) with Dunn’s post hoc correction, while normally distributed variables were analyzed by one-way analysis of variance (ANOVA), with Bonferroni correction for multiple comparisons. For ANOVA, the measured parameter was treated as the dependent variable and genotype or experimental condition as the independent variable. Statistical analyses were performed using GraphPad Prism 6 (GraphPad Software, San Diego, CA, USA), and figures were prepared using OriginPro 2021b (OriginLab Corporation, Northampton, MA, USA). Data are presented as Box-Plots or ± SEM and asterisks indicate statistical significance: ***p ≤ 0.0001, **p ≤ 0.001, and *p ≤ 0.01.

## Results

### Impaired Circadian Structural Remodeling of s-LN_v_’s in *RSK* Mutants

**Figure 1.**
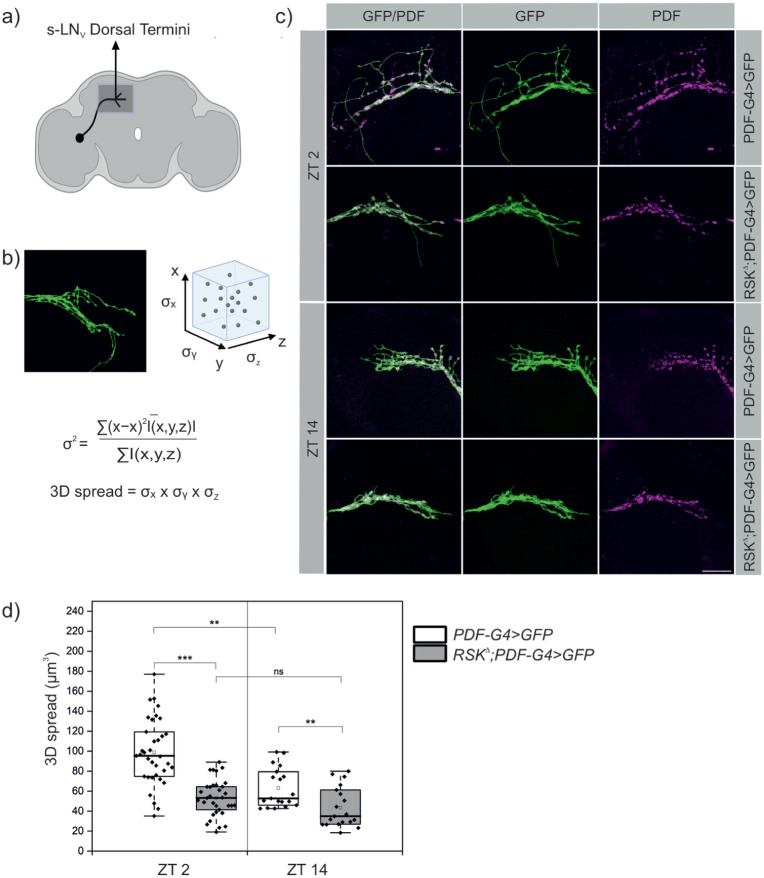
Loss of RSK disrupts daily structural changes of the s-LN_v_ dorsal projections. (a) Schematic representation of s-LN_v_ with their branched terminals in the dorsal protocerebrum (b) Analyzing the arborization complexity of s-LN_v_’s using the 3D spread method, which measures the spatial dispersion of the projections (Tassara, 2025). (c) Representative confocal images of s-LN_v_ projections from control and *RSK*^Δ^ flies dissected at ZT 2 and ZT 14 and then stained for endogenous PDF (magenta) and mCD8::GFP (green) expressed under PDF-Gal4 control. Scale bar: 20 μm. (d) Quantification revealed a significant reduction in the 3D spread of s-LN_v_ arborizations in *RSK*^Δ^ mutants relative to controls at both time points. The non-normally distributed data (Kolmogorov-Smirnov test; *p* < 0.02, Shapiro-Wilk test; *p* < 0.01) were analyzed using the Kruskal-Wallis test with Dunn’s correction for multiple comparisons.

The cell bodies of the four s-LN_v_’s in each hemisphere are localized laterally between the optic lobes and the central brain. A long neurite from each s-LN_v_ projects to the dorsal protocerebrum, where it turns medially and branches into several terminals (Figure 1a). The branching pattern undergoes circadian modulation with maximum spread at zeitgeber time (ZT) 2 in the early morning (open conformation) and a fasciculated state at ZT 14 (early night, closed conformation). Since previous studies have demonstrated a function of RSK in s-LN_v_ in regulating the circadian molecular oscillator (Akten et al., 2009; Beck et al., 2018) and kinases are ideally suited to transmit time-limited signals, we wanted to find out whether remodeling of s-LN_v_ terminals in the *RSK*^⊿^ null mutant is affected. Calculating the overall 3D spread (Figure 1b) of the terminal projections in wild-type animals confirmed previous studies with maximal branching at ZT 2 and the closed state at ZT 14 (Figure 1c and 1d). The *RSK*^⊿^ mutant showed a significant reduction in branching complexity at both time points when compared to wild type controls (Figure 1c and 1d). The effect was much more pronounced at ZT 2, indicating a function of RSK to promote branching (see also below).

However, this analysis measured the overall spread covered by the arborizations of all four s-LN_v_’s, leaving open the question, whether branching is also affected at the single cell level.

**Figure 2.**
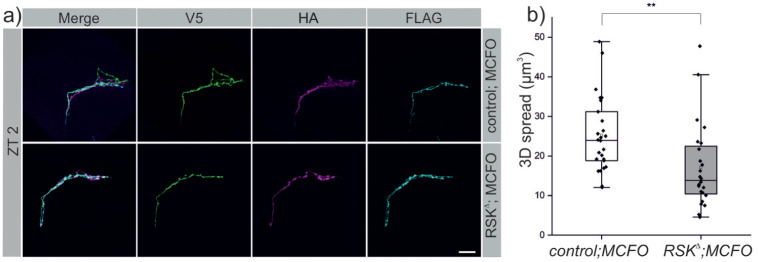
Loss of RSK reduces branching complexity of single s-LN_v_’s. (a) MultiColor FlpOut (MCFO) labeling of single s-LN_v_ by V5-, HA- or FlAG-tagged marker protein expression. Representative confocal images of the branching pattern of single stained s-LN_v_’s for control and *RSK*^Δ^ mutants dissected at ZT 2 are shown. Note the simple architecture of *RSK*^Δ^ s-LN_v_ projections with a main neurite that are, unlike in control animals, nearly devoid of side branches. Scale bar: 20 μm. (b) Quantification of the 3D spread at the level of single s-LN_v_’s. The non-normally distributed data (Kolmogorov-Smirnov test; *p* < 0.02, Shapiro-Wilk test; *p* < 0.008) were analyzed using the Mann-Whitney U test.

Recently it was shown that circadian changes in the s-LN_v_ branching pattern are not only a cycle of fasciculation and de-fasciculation of the arbors (Sivachenko et al., 2013), but in addition s-LN_v_’s add and lose axonal processes (Petsakou et al., 2015). To assess neuronal morphology at the single cell level, we marked individual s-LN_v_’s with the multicolor flipout (MCFO) technique at ZT 2, when complexity is maximal. In the controls, a main neurite can be seen from which one or more branches extend (Figure 2a). In contrast, the *RSK*^⊿^ mutant displayed a striking alteration in s-LN_v_ arbor morphology. Neurons showed a significant reduction in arbor complexity; in many cases only a single neurite without any branches was seen (Figure 2a). We also observed reduced medial extension of projections. 3D-spread analysis verified a significant difference between controls and *RSK*^⊿^ also at the single cell level (Figure 2b).

In summary, s-LN_v_ dorsal projections in the *RSK*^⊿^ mutant are not only locked in a fasciculated state but have also largely lost their cell-intrinsic property to increase branching complexity of terminals.

**Figure 3.**
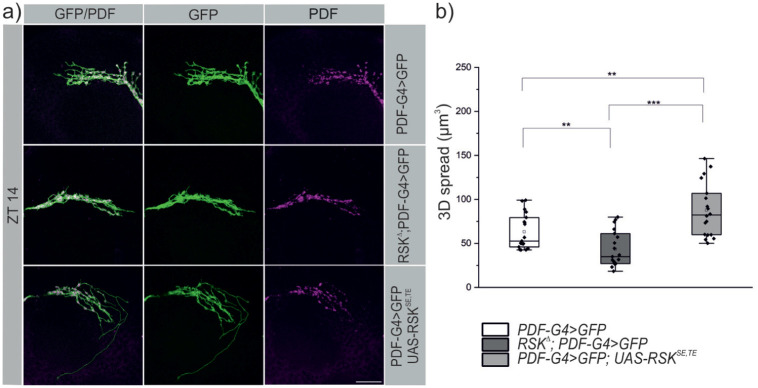
Activation of RSK forces s-LN_v_ terminals to adopt an open conformation at the wrong time of day. (a) Representative images of s-LN_v_ arborizations from control, *RSK*^Δ^ mutants, and flies expressing a constitutively activated RSK protein (RSK^SE,TE^) under *PDF-Gal4* control. All flies carry in addition a *UAS-mCD8::GFP* transgene. Stainings were performed at ZT 14 using antibodies against GFP (green) and endogenous PDF (magenta). In contrast to the fasciculated state in control and *RSK*^Δ^ flies (see also Figure 1), the sustained activation of RSK leads to increased branching complexity. Scale bar: 20 μm. (b) 3D spread and statistical analysis. The non-normally distributed data (Kolmogorov-Smirnov test; *p* < 0.02, Shapiro-Wilk test; *p* < 0.01) were analyzed using the Kruskal-Wallis test with Dunn’s correction for multiple comparisons.

Is RSK not only required but also sufficient in s-LN_v_’s to defasciculate their terminal projections? In other words, can we force s-LN_v_ projections to adopt the open conformation at ZT 14, where they are normally in the closed conformation? We assumed that RSK catalytic activities are necessary, but the extra- or intracellular signals that activate RSK via ERK in s-LN_v_’s are unknown. Therefore, we constitutively activated RSK by mimicking phosphorylation in the activation segment of the NTKD and CTKD through the combined substitution of serine 357 and threonine 732 with glutamic acid (S357E, T732E). Expression of the corresponding transgene *UAS-RSK*^SE,TE^ with *PDF-Gal4* in an otherwise wild-type background forced de-fasciculation of s-LN_v_ terminals at ZT 14 in comparison to the wild-type and *RSK*^⊿^ mutant controls (Figure 3a and 3b).

Apparently, s-LN_v_’s retain the principal ability to undergo structural changes throughout the day. The closed conformation seems to be the basal state. De-fasciculation is at least in part triggered by activation of RSK and mediated by so far unknown downstream effectors.

### Activation of Either RSK Kinase Domain Increases s-LN_v_ Branching Complexity

**Figure 4.**
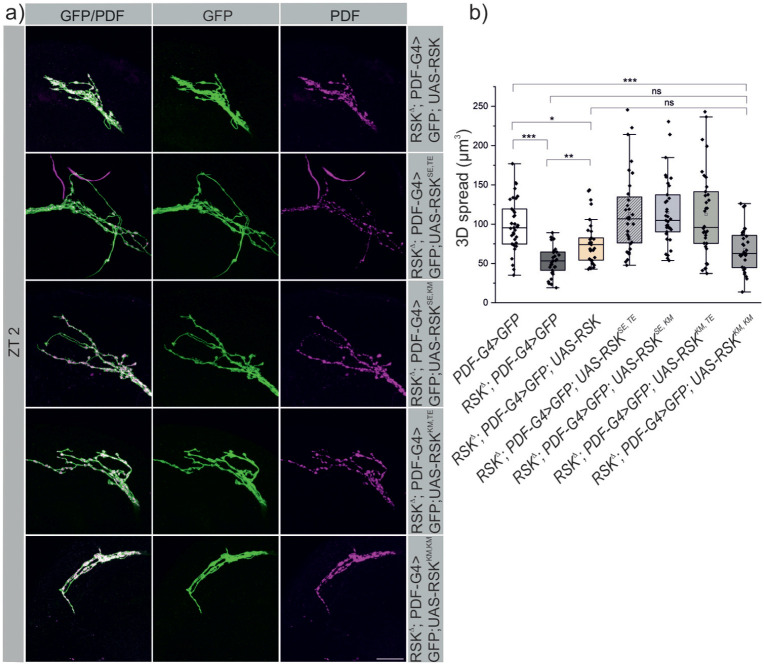
RSK function in s-LN_v_ remodeling requires both kinase domains. (a) The following *UAS-RSK* transgenes were expressed with *PDF-Gal4* in a *RSK*^Δ^, *UAS-mCD8::GFP* background: *UAS-RSK* (non-mutated), *UAS-RSK*^SE,TE^ (both kinase domains activated), *UAS-RSK*^SE,KM^ (NTKD active, CTKD inactive), *UAS-RSK*^KM,TE^ (NTKD inactive, CTKD active), *UAS-RSK*^KM,KM^ (both kinase domains inactive). Flies were dissected at ZT 2. Representative images of s-LN_v_ arborizations (stained for GFP and endogenous PDF) upon expression of each *RSK* transgene are shown. Scale bar: 20 μm. (b) 3D spread analysis, which in addition includes the data from the corresponding controls shown in Figure 1 (*PDF-Gal4; UAS-mCD8::GFP* and *RSK*^Δ^; *PDF-Gal4; UAS-mCD8::GFP*). Expression of non-mutated RSK resulted in partial but significant rescue, whereas inactivation of both kinase domains rendered the protein non-functional. Activation of each kinase alone or in combination completely rescued the *RSK*^Δ^ s-LN_v_ branching phenotype. The normally distributed data (Kolmogorov-Smirnov test; *p* > 0.2, Shapiro-Wilk test; *p* > 0.08) were analyzed by one-way ANOVA. Bonferroni correction was applied to account for multiple comparisons.

The common understanding is that the ERK-mediated activation of the CTKD triggers stimulation of the NTKD, which is responsible for phosphorylation of downstream substrates (Romeo et al., 2012). However, there are few exceptions. A NTKD deficient variant was still able to rescue the short period phenotype of the *RSK*^⊿^ mutant, whereas CTKD function is essential for RSK function in the circadian clock (Tangredi et al., 2012). To dissect the functional contribution of the NTKD and CTKD for s-LN_v_ neuronal remodeling, we analyzed transgenic flies carrying combinations of activating or inactivating mutations in both kinase domains. Specifically, we used a NTKD activated, CTKD inactivated variant (*UAS-RSK*^SE,KM^), a NTKD inactivated, CTKD activated variant (*UAS-RSK*^KM,TE^), both kinase domains activated (*UAS-RSK*^SE,TE^) respectively both kinase domains inactivated (*UAS-RSK*^KM,KM^) variants and a non-mutated transgene (*UAS-RSK)*. All transgenes were brought into a *RSK*^⊿^ mutant background and expressed in s-LN_v_’s using *PDF-Gal4*. 3D spread analysis was performed at ZT 2, the time point at which the s-LN_v_ terminals are in the open conformation in the wild-type but are closed in the *RSK*^⊿^ mutant (see Figure 1c). Compared to the *RSK*^⊿^ mutant, partial but significant rescue was observed with the non-mutated *UAS-RSK* transgene (Figure 4a and 4b). The completely kinase inactive variant RSK^KM,KM^ was unable to induce opening of s-LN_v_ terminals. The comparison between the non-mutated *RSK* transgene and the *RSK*^KM,KM^ transgene revealed only a tendency but no significant difference. Activation of both kinase domains (RSK^SE,TE^) or either kinase domain alone (RSK^SE,KM^, RSK^KM,TE^) was sufficient to fully restore the open conformation phenotype like in wild-type animals (Figure 4a and 4b). Several conclusions can be drawn from these findings. First, both kinase domains contribute independently to promote increased arborization complexity of s-LN_v_’s at ZT 2. Second, the mere presence of RSK in s-LN_v_ neurons is not sufficient; sustained activation of RSK is the crucial step to rescue the branching defect. Third, we consider two possibilities for the rather weak rescue ability of the wild-type RSK transgene. RSK might also be required in other cells, which in turn induce MAPK/ERK-mediated signaling and RSK activation in s-LN_v_’s. Alternatively, transgenic RSK protein levels deviate from endogenous RSK protein levels resulting in insufficient phosphorylation (activation) by ERK. The constitutively activated versions of RSK would overcome this limitation. We cannot address this question because antibodies against RSK and its phosphorylation status failed to work in stainings.

### Effect of *RSK*^Δ^ Mutants on BRP Accumulation in s-LN_v_ Terminals and PDF Expression

The dorsal aborizations of s-LN_v_’s harbor synaptic sites for bi-directional neurotransmitter-mediated communication with other neurons and PDF-filled dense core vesicles (DCV) for paracrine signaling (Helfrich-Förster and Reinhard, 2025).

**Figure 5.**
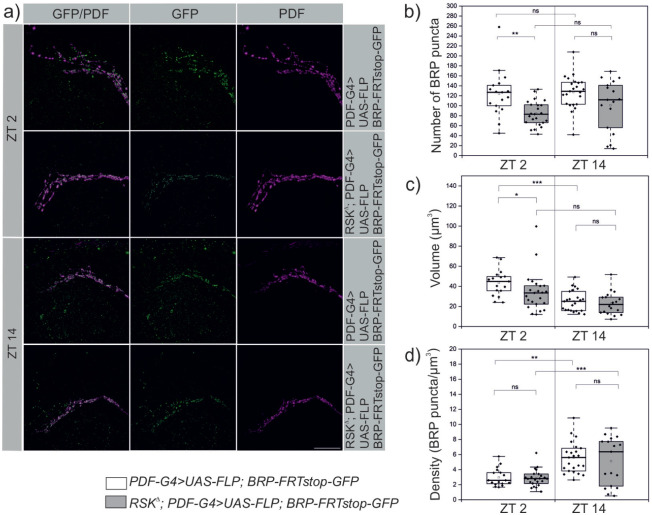
Quantification of BRP puncta in s-LN_v_ terminals using SIM microscopy. (a) *PDF-Gal4, UAS-Flp* induced removal of the FRT Stop cassette in *PBac{brp(FRT.stop)GFP}* allowed expression of BRP::GFP in s-LN_v_’s, which is spatially distinct from PDF signals as previously reported (Hofbauer et al., 2024). Scale bar: 20 μm. (b, c) Quantification of BRP puncta showed a significant decrease in *RSK*^Δ^ mutants only at ZT 2 with a corresponding decrease of the total s-LN_v_ terminal volume calculated from the PDF staining. (d) This resulted in unchanged density of BRP spots compared to controls. Because s-LN_v_ terminal volume is even more decreased in the *RSK*^Δ^ mutants at ZT 14 (b) without changes in BRP numbers (a), the density of BRP spots is increased like in controls (d). The normally distributed data for the number of BRP puncta and density (Kolmogorov-Smirnov test; *p* > 0.2, Shapiro-Wilk test; *p* > 0.08) were analyzed using one-way ANOVA with Bonferroni correction for multiple comparisons. Data for volume were non-normally distributed (Kolmogorov-Smirnov test; *p* < 0.04, Shapiro-Wilk test; *p* < 0.001) and were analyzed using the Kruskal-Wallis test with Dunn’s correction for multiple testing.

To test whether the failure in s-LN_v_ remodeling in *RSK*^⊿^ correlates with changes in synapse number, we quantified the presynaptic protein Bruchpilot (BRP) in s-LN_v_ terminals using the STaR method (Chen et al., 2014). It relies on expression of the GFP-tagged BRP protein (BRP::GFP) under its native promotor sequences upon cell type specific removal of a FRT stop cassette between the *BRP* and *GFP* encoding sequences. In our case, we used *PDF-Gal4* in combination with *UAS-Flippase*. With confocal miscroscopy it was difficult to clearly separate distinct BRP puncta, therefore higher resolution SIM microscopy was used (Figure 5a). Quantitative analysis revealed that the reduced branching complexity of s-LN_v_ terminals in the *RSK*^⊿^ mutant at ZT 2 resulted in a reduction in the absolute number of BRP puncta compared to the control (Figure 5b). Because the volume of the terminals was also decreased in the mutant (Figure 5c), the density of BRP spots was not changed compared to the controls (Figure 5d). At ZT 14, no significant differences between *RSK*^⊿^ mutants and controls were observed (Figure 5b-5d). Of note, the number of BRP puncta did not differ between ZT 2 and ZT 14 in controls. These results are consistent with an earlier study, which reported an unchanged number of BRP puncta between ZT 2 and ZT 14, but an increased density at time point ZT 14 (Hofbauer et al., 2024). However, they are not in agreement with other studies demonstrating a higher number of presynaptic sites at ZT 2 (Gorostiza et al., 2014; Ispizua et al., 2025). One explanation for this discrepancy could be that not all BRP spots detected by our method represent presynaptic sites but also include internalized BRP or BRP transport vesicles.

**Figure 6.**
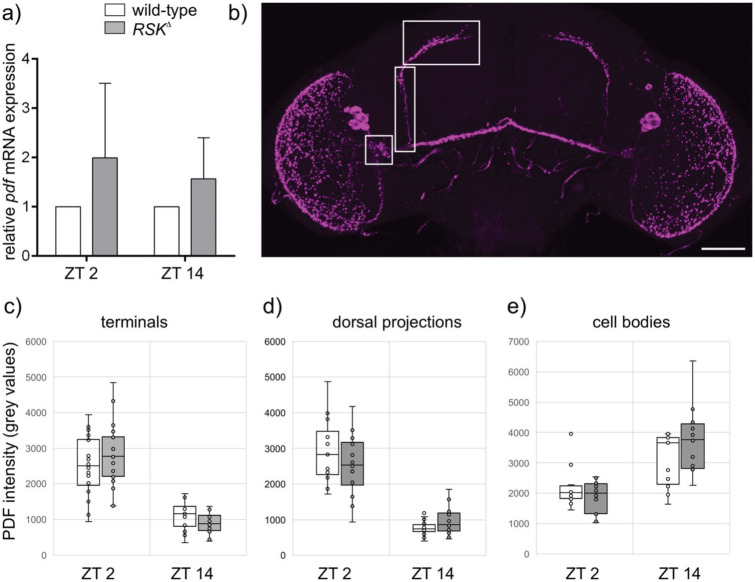
Loss of RSK has no influence on PDF expression. **(a)** RT-qPCR analysis from fly heads. Each bar represents the mean ± standard error of the mean (SEM) from at least four biological replicates, each repeated in triplicate. *PDF* mRNA expression levels were slightly higher in *RSK*^Δ^ mutants but not significantly different from wild-type. **(b-e)** The quantification of PDF protein expression levels in s-LN_v_ terminals **(c)**, dorsal projections **(d)**, and cell bodies **(e)** at ZT 2 and ZT 14 also showed no difference between *RSK*^Δ^ mutants and controls. The image in **(b)** shows the selected regions. Scale bar: 50 μm. For each time point and genotype, at least 20 hemibrains were analyzed.

PDF expression was analyzed at the transcriptional and peptide level. Previous studies showed that *pdf* gene expression remains constant throughout the day, while DCV packaged PDF accumulates before release by exocytosis in the morning (Klose et al., 2021; Park et al., 2000). Quantitative reverse transcription (RT)-PCR analysis confirmed that *pdf* mRNA levels in brains were not different between ZT 2 and ZT 14. Slightly increased, but not significantly different mRNA levels were measured in the *RSK*^⊿^ mutant at both time points (Figure 6a). For PDF peptide levels, signal intensities after staining with an anti-PDF antibody were quantified in s-LN_v_ cell bodies, along their dorsal projections and the branched terminals (Figure 6b), because PDF is detected in all these compartments and PDF output functions were maintained even in the absence of the terminal structures (Fernandez et al., 2020). PDF peptide levels in all three regions were not different between controls and *RSK*^⊿^ (Figure 6c-6e). Consistent with earlier findings (Klose et al., 2021; Park et al., 2000), PDF levels behaved in an opposite manner with highest expression in neurites and lowest expression in the cell bodies at ZT 2 and vice versa in the evening (Figure 6c-6e). This indicated that daily-regulated transport processes contribute to PDF signaling.

In summary, loss of RSK function has differential effects on s-LN_v_ output signaling. The number of BRP puncta was reduced at ZT 2, and together with the altered morphology, this could lead to changes in synaptic connectivity in the morning. PDF expression and distribution was unaffected with a tendency toward higher *pdf* expression levels (Figure 6a) and greater differences in PDF intensity between ZT 2 and ZT 14 in the s-LN_v_ cell bodies and terminals of *RSK*^⊿^ compared to wild-type flies (Figure 6c and 6e). However, we cannot rule the possibility that RSK influences transport dynamics, processing or signaling properties of PDF (Lee et al., 2023).

### Loss of RSK Affects Circadian Locomotor Activity and Photoperiod Adaptation

**Figure 7.**
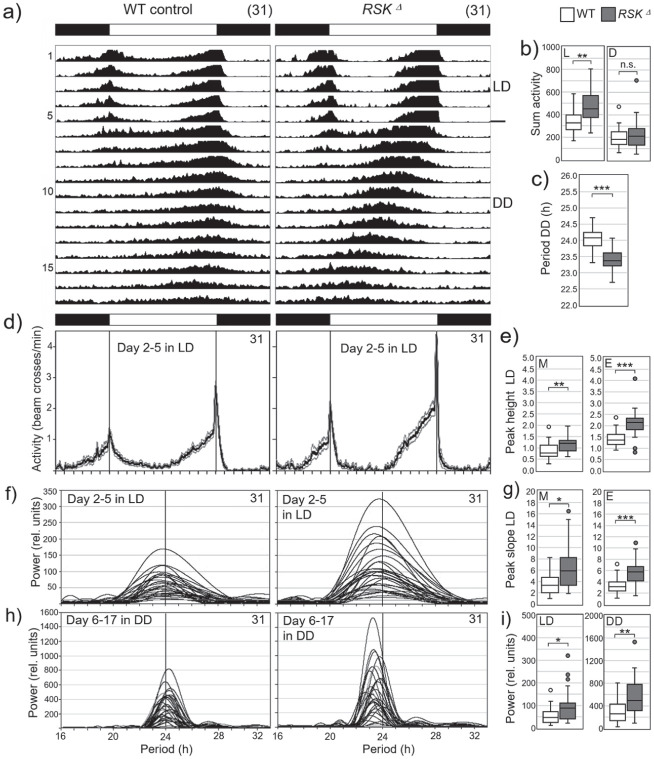
Activity rhythms of wild-type (WT) controls and *RSK*^Δ^ mutants under light-dark (LD) cycles and constant darkness (DD). **(a)** Average actograms of all 31 recorded flies per genotype. The flies were recorded for 5 days under LD and subsequently for 12 days under DD. Black and white bars on top indicate the dark and light portions of the LD cycle, respectively. **(b)** The mutants were significantly more active during the 12 h light part (L) of the day, but not during the 12 h dark part (D) of the day. **(c)** The free-running period under DD was significantly shorter in *RSK*^Δ^ mutants. **(d)** Average activity profiles of WT controls and *RSK*^Δ^ mutants (± SE) during day 2-5 in LD. *RSK*^Δ^ flies had higher morning (M) and evening (E) peaks than the WT controls **(e)** and steeper increases (slopes) of M and E activity **(g)**. **(f, h)** Superimposed periodograms indicating the rhythm period and power of all flies under LD and DD. The gray lines in the periodograms indicate the significance level of *p* = 0.05. **(i)** Calculated medians of rhythm power under LD and DD. *RSK*^Δ^ mutants had a significantly higher rhythm power than the WT controls under both conditions. The Mann-Whitney U test was used for pairwise comparisons.

Previous studies have established the function of RSK as a regulator of the molecular circadian oscillator (Akten et al., 2009; Beck et al., 2018). The originally used *RSK* loss-of-function mutant (*RSK*^Δ58/1^) showed a short period phenotype under constant darkness conditions (DD), a phenotype also confirmed by the new *RSK*^⊿^ mutant used in this study (Figure 7a and 7c). A careful analysis of the locomotor activities uncovered several novel phenotypes. Like wild-type flies, *RSK*^⊿^ flies showed low activity during siesta and at night, but during the day their activity rose faster and to higher levels than wild-type flies in the morning and evening (Figure 7d, 7e, 7g), resulting in rhythms with high amplitude and high power (Figure 7f and 7i). These rhythms with high amplitude and power continued in DD (Figure 7h) as can be seen in the power of the free-running rhythms, which was significantly higher in *RSK*^⊿^ mutants than in wild-type flies (Figure 7i).

Overall, *RSK*^⊿^ mutants exhibited very stable and robust circadian rhythms that could result in less sensitivity to disturbances than those of wild-type flies. This hypothesis was supported by an experiment in DD in which the temperature control in the incubator failed and the temperature initially rose to 35°C and then dropped to ~16°C until it stabilized again (red shaded area in the average actograms in Suppl. Fig. S1). While most wild-type flies developed complex rhythms or even became arrhythmic after this event, all *RSK*^⊿^ mutants remained rhythmic and continued to free-run with significantly higher power than their wild-type siblings (Suppl. Fig. S1).

**Figure 8.**
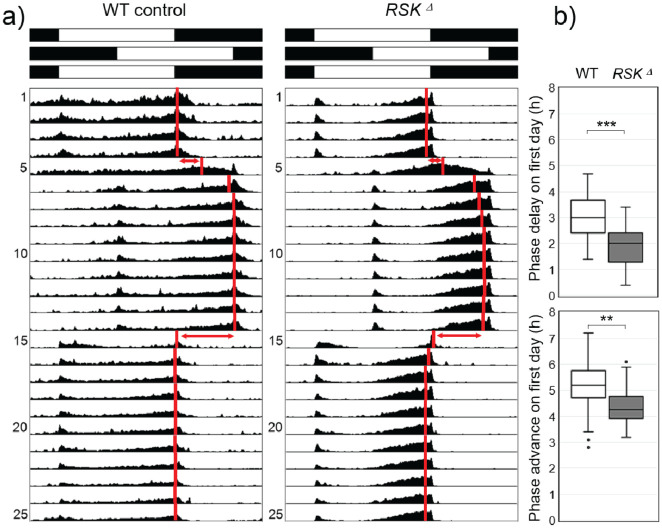
Activity rhythms of wild-type (WT) controls and *RSK*^Δ^ mutants under light-dark (LD) cycles, which were phase delayed and advanced. (a) Average actograms of all 32 recorded flies per genotype. Black and white bars on top indicate the sequence of the 6-hour phase shifts. Red vertical bars indicate the calculated mean phases of the evening activity peaks. (b) Accomplished phase shifts on the first day of the shifted LD cycles. The Mann-Whitney U test was used for pairwise comparisons.

There is a reciprocal relationship between the amplitude (robustness) of an oscillator (clock) and its ability to shift its phase (Pulivarthy et al., 2007). Robust clocks with high amplitude take longer than weak clocks with low amplitude to adapt to phase shifts of the light-dark cycle in so-called “jet lag” experiments (Ananthasubramaniam et al., 2020; Vitaterna et al., 2006). To further test whether *RSK*^⊿^ flies have more robust clocks than wild-type flies, we conducted such a jet lag experiment, in which we first delayed the light-dark cycle by 6 hours and then advanced it back to its original phase 10 days later (Figure 8a). As reported previously (Helfrich-Förster et al., 2001) wild-type flies re-entrained their activity very quickly to the shifted LD cycles (Figure 8a). The *RSK*^⊿^ flies took slightly longer than the wild-type flies to undergo phase shift. In particular, their phase delays and phase advances on the first days after the shifts were significantly smaller than those of the wild-type flies (Figure 8b). This finding confirms that they have stronger clocks than wild-type flies.

**Figure 9.**
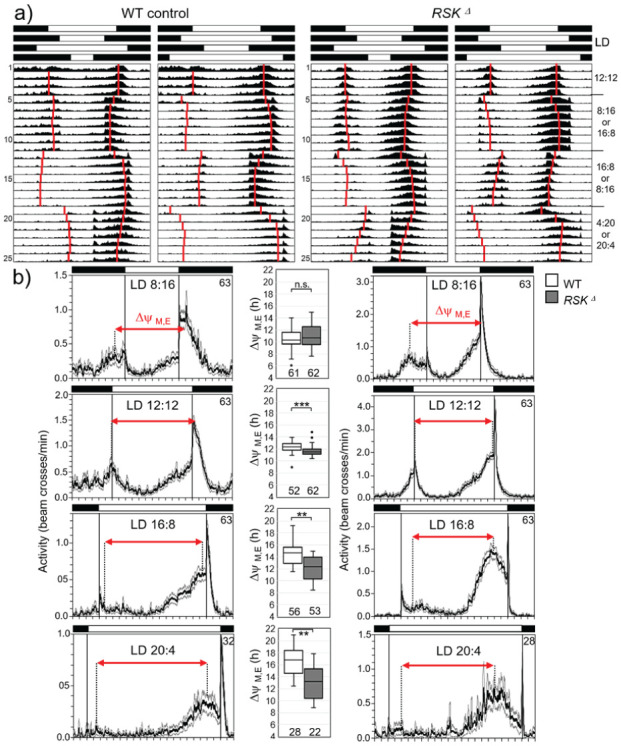
Activity rhythms of wild-type (WT) controls and *RSK*^D^ mutants under light-dark (LD) cycles with different photoperiod. (a) Average actograms of all 32 recorded flies per genotype. Black and white bars on top indicate the sequence of the different photoperiods, respectively. Note that the activity scales of wild-type (WT) controls and *RSK*^Δ^ mutants are different. Red vertical bars indicate the calculated mean phases of morning and evening activity peaks. (b) Average activity profiles of WT controls and *RSK*^Δ^ mutants (± SE) for the last 2 days under the different photoperiods (except for LD4:20 under which no stable phase was reached until the end of recording). The phase relationship between morning and evening peaks (Δψ_M,E_) is indicated as red arrow in the average activity profiles and shown for WT controls and *RSK*^Δ^ mutants in box plots between the relevant activity profiles. Some flies lacked the morning activity bout. Therefore, the numbers of flies for which morning and evening peaks could be determined and Δψ_M,E_ be calculated (indicated below the boxplots) is lower than the number of recorded flies. The Mann-Whitney U test was used for pairwise comparisons.

In nature, flies are not exposed to sudden phase shifts in the light-dark cycle, but in temperate regions they experience significant changes in day length (photoperiods) in spring and fall, to which they must adapt. Flies do so by tracking dawn and dusk (or lights-on and lights-off) with their morning and evening activities, respectively (Rieger et al., 2012). Previous studies have shown that flies with weak clocks are better able to adapt to extreme photoperiods than flies with more robust clocks (Beauchamp et al., 2018; Manoli et al., 2025; Menegazzi et al., 2017; Vaze et al., 2024). Here, we tested the ability of *RSK*^⊿^ and wild-type flies to adapt to sudden quite dramatic changes in photoperiod. When entrained under 12:12-hour LD conditions and then shifted to long-day (16:8 LD) or short day (8:16 LD) conditions, wild-type flies adapted their morning and evening activity peaks very quickly to the new light on/off regime. Even direct switching from long to short days or vice versa took place without delay in adjustment (Figure 9a). In contrast, *RSK*^⊿^ flies needed several days to gradually adjust their activity rhythm to the new light regime, regardless of the order in which long and short days followed each other (Figure 9a). In addition, *RSK*^⊿^ flies were not able to extend the distance between morning and evening activity peaks (phase-relationship Δψ_M, E_) beyond 13 hours, which was significantly less than found in wild-type flies, who could achieve a Δψ_M, E_ of 17 hours (Figure 9b). This shows that the circadian clock of *RSK*^⊿^ flies is not only stronger but at the same time less plastic than the wild-type clock, which makes entrainment to changing photoperiods more difficult.

In summary, the robust clock of *RSK*^⊿^ mutants comes at the expense of flexibility in responding to changing light conditions.

## Discussion

### Role of the s-LN_v_’s in the Clock Network

Within the clock neuron network, the s-LN_v_’s have a central function (Top and Young, 2018). Their presence and electrical activity are essential for the flies’ circadian rhythmicity (Helfrich-Förster, 1998; Nitabach et al., 2002; Stoleru et al., 2004), and a functional clock only in them in an otherwise *per*^0^ background is sufficient to provoke morning activity and circadian rhythmicity (Grima et al., 2004; Rieger et al., 2009; Sekiguchi et al., 2024). Although other clock neurons can compensate for the loss of a functional clock in the s-LN_v_’s (Delventhal et al., 2019; Schlichting et al., 2019), and recent studies demonstrated that a functional clock in certain dorsal clock neurons is sufficient for morning and evening activity and circadian rhythmicity under DD conditions (Reinhard et al., 2025; Sekiguchi et al., 2024), this was only possible in the presence of PDF (Reinhard et al., 2025).

### Role of PDF

PDF is the main output from s-LN_v_’s and it is essential to regulate circadian behavior (Renn et al., 1999). Accumulation and release of PDF in s-LN_v_ is clock-controlled (Park et al., 2000) and peaks in the morning (Klose et al., 2021). PDF seems to be important for appropriate behavior as all manipulations of the s-LN_v_’s that caused rhythm defects were associated with absent or permanent low PDF levels in s-LN_v_ terminals (Depetris-Chauvin et al., 2011; Duhart et al., 2020; Fernandez et al., 2007; Hodge and Stanewsky, 2008). To generate the functional neuropeptide from the proPDF precursor, several posttranslational modifications are required, including cleavage and amidation (Lee et al., 2023). Although we did not observe significant changes in transcription and rhythmic accumulation of PDF in s-LN_v_’s of *RSK*^⊿^ mutants compared to wild-type, a potential function of RSK in proPDF processing, axonal transport, release or signaling properties of mature PDF cannot be ruled out. *PDF* mutants are strongly arrhythmic (Renn et al., 1999) and proPDF processing is an essential step for normal PDF dependent rhythmicity (Lee et al., 2023) whereas *RSK*^⊿^ flies exhibit strong rhythmicity. Therefore, we consider it unlikely that RSK influences the signaling properties of PDF, but it warrants detailed analysis in view of the observed anterograde axonal transport defects seen in motor neurons of *RSK* mutants (Beck et al., 2015).

### Role of Circadian Remodeling

The circadian remodeling of the s-LN_v_ terminals is under control of the circadian clock (Fernandez et al., 2008) and dependent on electrical activity (Depetris-Chauvin et al., 2011). At least one study suggests that circadian remodeling of the s-LN_v_ terminals is also essential for circadian rhythmicity (Petsakou et al., 2015). In this study, the RhoGTPase Rho1 was overexpressed in the s-LN_v_’s, which provoked a lock of the s-LN_v_ terminals in the closed conformation and lead to arrhythmic behavior. Rho1 is a main regulator of the actin cytoskeleton dynamics, and its activity is clock-controlled via rhythmic transcription of the guanine nucleotide exchange factor Pura (Petsakou et al., 2015). Rho1 activity is highest at dusk leading to retraction of s-LN_v_ terminals. Consequently, Rho1 overexpression keeps the terminals in the closed conformation, but it is not clear whether Rho1 overexpression simultaneously affected PDF accumulation and release.

### PDF Expression and Remodeling of the s-LN_v_ Termini Are Independent Events

Both PDF accumulation and the daily remodeling of the s-LN_v_ termini appear to contribute to the control of rhythmic activity patterns, and it has been unclear whether the two events are dependent on each other. Here, we clearly show that they are independent of each other: *RSK*^⊿^ flies lack the daily remodeling of the s-LN_v_ termini but retain rhythmic PDF accumulation and circadian rhythmicity. This indicates that the daily remodeling of the s-LN_v_ termini is not essential for circadian rhythmicity, and this is consistent with earlier reports. Fernandez et al. (2020) expressed the repulsive axon guidance receptor unc-5 in the s-LN_v_’s, which completely prevented formation of terminal structures. Nevertheless, behavioral circadian rhythmicity was not affected, which suggests that rhythmic PDF release was normal and does not depend on the presence of the terminals (Fernandez et al., 2020). Indeed, PDF is released from dense core vesicles by paracrine signaling and such release sites are spatially separated from presynaptic zones (Hofbauer et al., 2024) and distributed all along the axons (Klose et al., 2021). Furthermore, the downregulation of BRP and other active zone proteins in the s-LN_v_’s did not affect PDF-dependent circadian rhythmicity (Hofbauer et al., 2024). Altogether, this strongly suggests that the synapses in the terminals are neither needed for rhythmic PDF release nor important for circadian rhythmicity.

### Putative Importance of s-LN_v_ Plasticity for Behavioral Rhythms

The daily remodeling of the s-LN_v_ terminals most likely affects the synaptic contacts between the s-LN_v_’s and other neurons, and by this way provokes plasticity in rhythmic behavior. Although the s-LN_v_’s form not many synaptic contacts with other neurons (Reinhard et al., 2024; Shafer et al., 2022), the s-LN_v_ terminals contain few presynaptic and postsynaptic sites (Yasuyama and Meinertzhagen, 2010), suggesting that failure in remodeling could affect synaptic in- and output communication.

#### Output Communication

The only neurotransmitter detected in the s-LN_v_ so far is the inhibitory neurotransmitter glycine, which seems to signal to several other clock neurons via three different GABA/glycine receptor subunits (Frenkel et al., 2017). Most interestingly, knocking down single or all three receptor subunits in most clock neurons (with *Clk856-Gal4*), shortened the flies’ circadian period and increased rhythm power (Frenkel et al., 2017), strongly reminding on the phenotype of the *RSK*^⊿^ flies. Here, we found that the number of presynaptic sites is reduced in *RSK*^⊿^ at ZT 2, suggesting less inhibitory output to the postsynaptic clock neurons in the morning. Possibly this can cause high-power rhythms and contribute to the short-period of *RSK*^⊿^ flies, which was explained so far solely by the interaction of RSK with Shaggy (SGG) in the clock neurons (Beck et al., 2018). Changes in period can also be caused by genetic manipulation of neurons downstream the clock neurons (Scholz-Carlson et al., 2025). The s-LN_v_’s form strong synaptic connections with three neurons in the superior lateral protocerebrum, called SLP316. It is not yet known whether the SLP316’s express glycine receptors but silencing or hyper-exciting them changes the free-running period of the flies in a reciprocal manner without reducing rhythmicity (Scholz-Carlson et al., 2025). The three SLP316’s signal to DN1_p_, clock neurons that play a major role in the control of sleep and activity (Lamaze and Stanewsky, 2019). Consequently, the SLP316’s are an important component of the wider clock network, and their neuronal activity, which might increase after less inhibitory input from the s-LN_v_’s, can affect core clock parameters like period and rhythm power.

#### Input Communication

The s-LN_v_’s receive sparse synaptic input from glutaminergic and cholinergic neurons (Reinhard et al., 2024), which appears important for transferring environmental signals to them. Sensing environmental information in a proper way and may depend on the daily remodeling of input synapses, which then allows the animals to adapt to environmental changes. The idea that the daily s-LN_v_ remodeling is necessary for adaptation of the circadian clock to different environmental conditions is not new. Fernandez et al. (2020) showed that the loss of s-LN_v_ termini causes deficits in the behavioral entrainment to naturalistic temperature cycles, and like us, Petsakou et al. (2015) found that structural s-LN_v_ plasticity is important for seasonal adaptation to short- and long-days. Thus, the daily terminal remodeling may enhance behavioral plasticity, which helps adapting to changing environmental conditions.

#### Reciprocal Communication Between Clock Neurons

As daily synaptic plasticity is not restricted to the s-LN_v_’s but also present in other clock neurons, the reciprocal communication between different clock neurons might be affected in *RSK*^⊿^ flies. For example, the dendrites of DN1_a_ neurons synapse with the s-LN_v_ dorsal branches whereas DN1_a_ axons terminate close to the s-LN_v_ somata (Song et al., 2021). Daily remodeling of these neurons is antiphasic such that the maximum spread of DN1_a_ axon terminals coincides with the closed conformation of s-LN_v_ terminals and vice versa. It has been proposed that these daily shifting connectivity between s-LN_v_’s and DN1_a_’s contribute to generating predictions about light changes (Song et al., 2021). A second reciprocal remodeling exists between LN_d_’s, as members of the so-called evening cells, and s-LN_v_’s (morning cells). Inhibition of the excitatory cholinergic feedback from LN_d_’s to s-LN_v_’s leads to reduced rhythmic power and low non-cycling levels of PDF in s-LN_v_ terminals (Duhart et al., 2020).

Regarding RSK, the glutamatergic feedback inhibition from DN1_p_’s to s-LN_v_’s and LN_d_’s could be interesting for two reasons. On one hand, blocking feedback inhibition increases the activity of flies without affecting free-running rhythmicity (Guo et al., 2016); on the other hand, studies in hippocampal neurons of vertebrates and at the neuromuscular junction in *Drosophila* demonstrated a role for RSK in glutamatergic neurotransmission (Beck et al., 2015; Mehmood et al., 2011; Morice et al., 2013; Schneider et al., 2011). Therefore, one explanation for the enhanced morning and evening activities of *RSK*^⊿^ flies could be disturbance of glutamatergic feedback inhibition either by changes in signal transmission and/or by alterations in synaptic connectivity with DN1_p_’s.

### RSK Function in Neuronal Remodeling

Our findings highlighted a critical role for RSK kinase activity in neuronal remodeling. Given the prevailing opinion on the sequential activation mechanism of RSK with the N-terminal kinase domain as the main effector kinase, the independent requirement of both kinase domains appears surprising at first glance. Yet, the importance of analyzing the function of RSK in a cell type-specific context became evident by several studies in *Drosophila*. RSK function in the molecular circadian oscillator is independent of NTKD activity but requires a functional CTKD (Beck et al., 2018; Tangredi et al., 2012). Even more unusual, during eye development, RSK serves as a cytoplasmic anchor for ERK, independent of kinase activity (Kim et al., 2006). Therefore, to understand RSK function in neuronal remodeling, identification of molecular targets is urgently needed. Our analysis at the cell population and single cell level uncovered two distinct effects. First, the terminals remain fasciculated. This indicated a requirement of RSK for regulation of intercellular cell adhesion. Second, fewer side branches extend from the main branch of a single s-LN_v_. RSK might interfere with cytoskeletal dynamics, neurite growth or guidance, processes that play an important role in s-LN_v_ terminal structure formation (Fernandez et al., 2020; Petsakou et al., 2015). Based on these phenotypes, potential candidates include cell adhesion molecules to regulate intercellular fasciculation of neurites, molecules mediating intracellular self-avoidance of neurite branches and proteins regulating outgrowth or disassembly of neurite branches. Proximity biotin labeling by expression of a RSK-TurboID construct in neurons identified proteins working in each these processes (A.T.M. and T.R., unpublished data), yet the direct interaction and functional relevance in s-LN_v_’s remains to be verified in future work. Another open question of our study is whether loss of RSK not only affects neuronal remodeling of s-LN_v_ terminals but also has an impact on expression, transport or delivery of synaptic proteins and their assembly to higher-order structures. In *Drosophila* motoneurons, loss of RSK resulted in accumulation of the presynaptic scaffold protein BRP and the synaptic vesicle associated cysteine string protein (CSP) in the proximal part of the axon and in impaired anterograde mitochondrial transport (Beck et al., 2015). Although the density of BRP in s-LN_v_ terminals was not altered in *RSK*^⊿^ mutants, one should use ultrastructural techniques (Ispizua et al., 2025) to analyze synaptic organization in more detail. This relates to the finding that loss of RSK2 in mice does not affect the density of excitatory synapses in hippocampal neurons but modifies their morphology, which might alter synaptic transmission (Morice et al., 2013).

In summary, our findings add a new layer of complexity to the function of RSK in the nervous system. In addition to its known role in glutamatergic neurotransmission, time-of-day-dependent neural remodeling may also influence neural circuitry. Disruption of both processes in RSK mutants could contribute to the pathophysiology and behavioral impairments associated with Coffin-Lowry syndrome.

## Supplemental Material

sj-pdf-1-jbr-10.1177_07487304261434715 – Supplemental material for Drosophila RSK: A Pivotal Regulator of Circadian Plasticity at the Neuronal and Behavioral Level Supplemental material, sj-pdf-1-jbr-10.1177_07487304261434715 for Drosophila RSK: A Pivotal Regulator of Circadian Plasticity at the Neuronal and Behavioral Level by Athira Theyyassanchery Mani, Vivian Backs, Christian Werner, Charlotte Helfrich-Förster and Thomas Raabe in Journal of Biological Rhythms
