# Supplementary material for: Drosophila RSK: A Pivotal Regulator of Circadian Plasticity at the Neuronal and Behavioral Level
Source: J Biol Rhythms. 2026 Apr 12;41(4):416–34. doi: 10.1177/07487304261434715 (PMC13342480; doi:10.1177/07487304261434715)
Supplement: sj-pdf-1-jbr-10.1177_07487304261434715 – Supplemental material for Drosophila RSK: A Pivotal Regulator of Circadian Plasticity at the Neuronal and Behavioral Level [file sj-pdf-1-jbr-10.1177_07487304261434715.pdf]

Supplementary Material

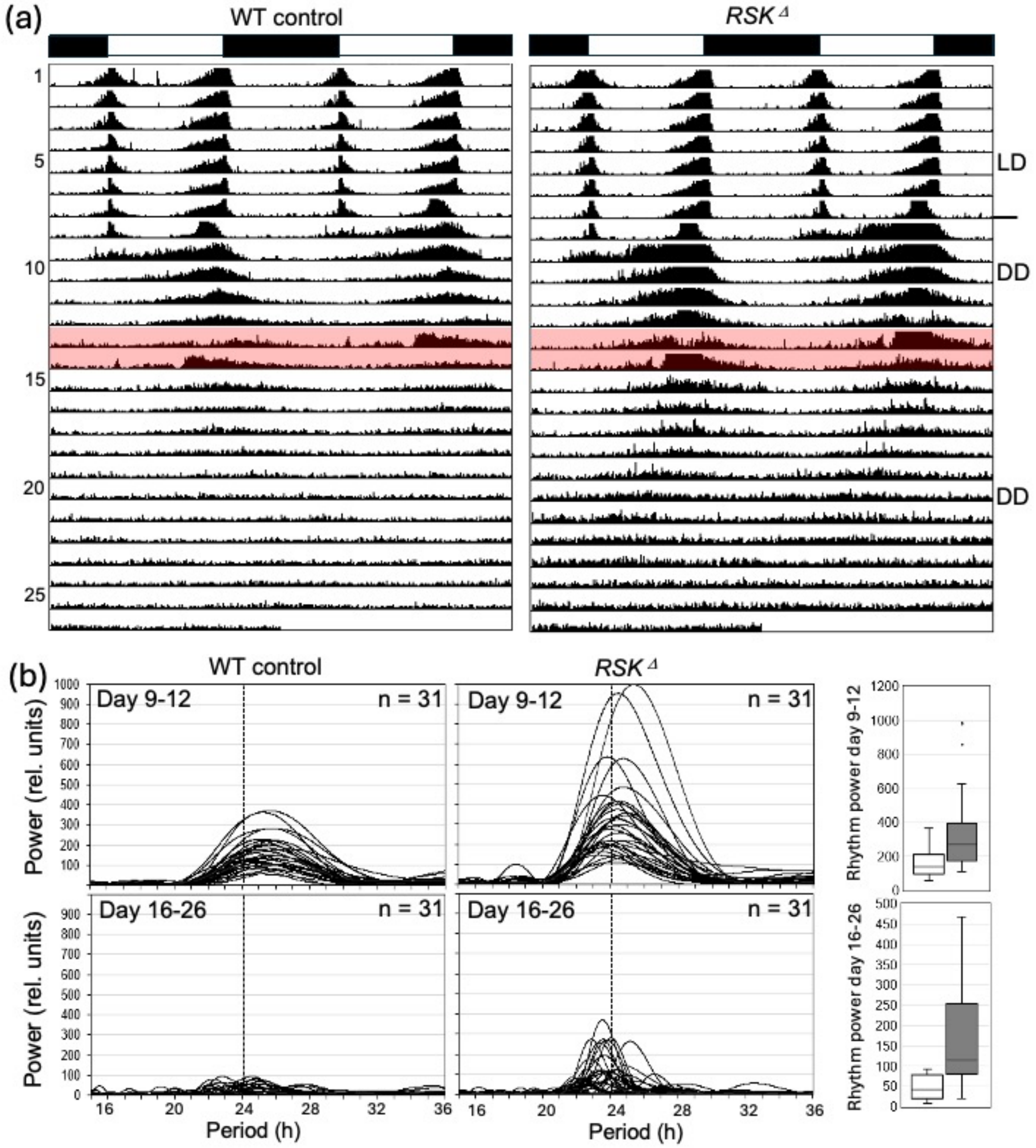

Supplementary Figure S1. Activity rhythms of wild-type controls (WT) and *RSK*$^{\Delta}$ mutants under light-dark cycles (LD) and constant darkness (DD) with a failure of temperature control on days 13–14.

(a) Average actograms (double plots) of all 31 recorded flies per genotype. The flies were recorded for 8 days under LD and then for 18 days under DD. The black and white bars at the top indicate the dark and light periods of the LD cycle, respectively. On days 13–14, the temperature control in the chamber failed and the temperature rose to 35 °C; it then dropped to ~16 °C until it stabilized again at 20 °C. **(b)** Superimposed periodograms and calculated medians of rhythm power (right box plots) before and after the temperature control failure. The *RSK*$^{\Delta}$ mutants had higher rhythm power than the WT controls, and their rhythms were less affected by the temperature disturbance.
